# Systematic review of diarrhea duration and severity in children and adults in low- and middle-income countries

**DOI:** 10.1186/1471-2458-12-276

**Published:** 2012-04-06

**Authors:** Laura M Lamberti, Christa L Fischer Walker, Robert E Black

**Affiliations:** 1 Department of International Health, Johns Hopkins Bloomberg School of Public Health, 615 N. Wolfe St, Baltimore MD 21205, USA

## Abstract

**Background:**

Diarrhea is a leading cause of morbidity and mortality globally; yet the overall burden of diarrhea in terms of duration and severity has not been quantified. As improvements in treatment lead to decreases in diarrhea mortality, it is important to understand the substantial impact of diarrhea morbidity on disability among children and adults worldwide.

**Methods:**

We conducted a systematic review to generate estimates of duration and severity outcomes for individuals 0-59 mos, 5-15 yrs, and ≥ 16 yrs, and for 3 severity indexes: mild, moderate, and severe.

**Results:**

We estimate that among children under-five, 64.8% of diarrheal episodes are mild, 34.7% are moderate, and 0.5% are severe. On average, mild episodes last 4.3 days, and severe episodes last 8.4 days and cause dehydration in 84.6% of cases. We estimate that among older children and adults, 95% of episodes are mild; 4.95% are moderate; and 0.05% are severe. Among individuals ≥ 16 yrs, severe episodes typically last 2.6 days and cause dehydration in 92.8% of cases.

**Conclusions:**

Moderate and severe episodes constitute a substantial portion of the total envelope of diarrhea among children under-five (35.2%; about 588 million episodes). Among older children and adults, moderate and severe episodes account for a much smaller proportion of the total envelope of diarrhea (5%), but the absolute number of such episodes is noteworthy (about 21.5 million episodes among individuals ≥ 16 yrs). Hence, the global burden of diarrhea consists of significant morbidity, extending beyond episodes progressing to death.

## Background

Diarrhea is a leading cause of morbidity and mortality across all age groups and regions of the world. Among children 0-59 months of age, diarrhea is responsible for 1.236 million deaths annually and is the second leading cause of death in this age group [1]. Though mortality rates among older children, adolescents, and adults are lower than those observed in children under five, diarrhea still poses a substantial burden accounting for approximately 2.8 billion diarrhea episodes among older children, adolescents, and adults [2].

Given the importance of diarrhea as one of the foremost causes of global morbidity and mortality, it is essential to quantify factors, such as duration and severity, critical to quantifying the overall burden of disease attributable to diarrhea. Comprehensive estimates of diarrheal duration and severity will allow for the more accurate assignment of disability adjusted life years (DALYs) in terms of years lost to disability due to diarrheal illness. While various studies conducted throughout the world report on the duration and severity of diarrhea, comprehensive estimates do not exist in the literature. Furthermore, there is a lack of consistency in methods for defining severe, moderate, and mild diarrheal episodes despite the existence of severity scales, such as the Hjelt, Vesikari, and Clark scoring systems, which assign an overall degree of severity based on the sum presentation of symptoms [3-5]. We therefore conducted a systematic literature review with the goal of consolidating available data on the duration and severity of diarrhea and describing the overall burden of episodes among children and adults in low- and middle-income countries.

## Methods

### Search strategy and selection criteria

We systematically reviewed all literature published from 1990 to 2010 to identify studies reporting measures of diarrhea duration and severity in children and adults. From May 20-27, 2010, we searched in Pubmed using combinations of MeSH search terms: diarrhea, gastroenteritis, duration, persistence, severity, infant, child, teenage, and adult.

We initially screened all unique publications for eligibility based on the relevancy of title and abstract; we then screened the full manuscripts for inclusion and exclusion criteria. We included randomized controlled trials (RCTs), cohort, and observational studies published in any language and conducted in any country. Included studies contained data on diarrhea duration and/or severity in children and/or adults. We excluded studies with unclear methodology and diarrhea recall beyond 2 weeks. We accepted studies defining diarrhea as the passage of ≥ 3 watery stools in a 24-hour period; we also considered mother's report of a change in usual stool consistency or frequency for infants ≤ 11 mo of age, and the diagnosis of diarrhea by a physician or nurse as valid case definitions. We excluded studies describing diarrhea deaths alone since duration and severity outcomes of episodes resulting in death are not generalizable to all diarrhea episodes. We also excluded reports of nosocomial outbreaks, diarrhea due to known chronic or non-infectious illness, and studies that limited inclusion to one etiologic agent. For analytical purposes, we did not include studies that combined outcomes across child/adult age categories or inpatient/outpatient status. Furthermore, for duration outcomes, we included studies on the natural course of diarrhea episodes and excluded those reporting on acute or persistent episodes alone.

### Data abstraction

We abstracted measures of diarrhea duration and severity for all ages; for presentation purposes we grouped these data into 3 distinct age categories: 0-59 mos, 5-15 yrs, and ≥ 16 years of age. For studies stratifying outcomes by treatment or HIV-status, we only abstracted data on placebo and HIV-negative individuals, respectively.

We abstracted three outcomes describing diarrhea duration: mean or median duration--reported as the number of days per episode of diarrhea, and the proportion of total diarrheal episodes that became persistent (≥ 14 or ≥ 15 days). If the proportion of persistent cases was skewed due to an intentional sampling bias or matching, we did not include the study for this outcome. To ensure capture of the full length of diarrheal episodes, we also excluded studies solely reporting the duration of diarrheal episodes at baseline.

Multiple outcomes were reported as measures of severity. We abstracted the proportion of individuals suffering from diarrhea with mild, moderate, severe, or any dehydration; vomiting; and bloody stools. We classified necessitating ORS or intravenous fluids as any dehydration. For studies assessing dehydration by both physician perception and WHO classification for dehydration, we utilized the latter. Likewise, if possible, we abstracted outcomes assessed by physicians or trained health workers in place of those estimated by mothers or caregivers. Additional severity outcomes included the mean stool volume (g/kg/day) and mean stool frequency (stools/day). We also abstracted the proportion with low (< 40 g/kg/day), medium (40-70 g/kg/day), and high (> 70 g/kg/day) stool volume. For stool frequency, we abstracted the proportion low (1-5 stools/day), medium (6-9 stools/day), and high (≥ 10 stools/day), as well as the proportion with > 5 stools/day and > 3 stools/day.

We did not find any studies able to quantify the proportion of episodes that remain mild/moderate or that progress to severe. We therefore used care-seeking behavior among caregivers of children < 5 years of age as a proxy for diarrhea severity in this age group. We abstracted care-seeking behavior from the Demographic Health Surveys (DHS) [6]. The DHS reports the number of children ≤ 35 months of age with diarrhea in the two weeks preceding the survey for whom care was sought, as well as the number who were not taken to a health provider.

### Data analysis

We used STATA 10.1 to generate combined estimates and 95% confidence intervals for all duration and severity outcomes [7]. To ensure that confidence intervals were lower bound by zero for all outcomes and upper bound by one for proportions, we fit a logistic regression model to data reported as proportions and a Poisson regression model to continuous outcomes. All outcomes were weighted by sample size, which consisted of the total number of episodes. For study designs assessing only one episode per individual, the number of children or adults with diarrhea was considered the total number of episodes. We used the reported diarrheal incidence (number of episodes per child-year) to estimate the sample size for studies evaluating multiple episodes per child without reporting the total number of episodes. We conducted separate analyses for each study type--community, hospital-based inpatient and hospital-based outpatient.

Using the DHS data, we calculated region-specific averages for diarrhea care-seeking. We also calculated the median global estimate of diarrhea care-seeking across regions. The global estimate was used to inform our model describing the total envelope of diarrhea among children under five. Mild cases were defined as those for which no care was sought, and moderate cases were defined as those for which care was sought through a health provider. We assumed that mild and moderate episodes were best described by the duration and severity outcomes derived from community-based studies and hospital outpatient studies, respectively. We used the proportion of outpatient cases presenting with severe dehydration to determine the percentage of moderate cases which progress to severe. Duration and severity outcomes derived from hospital inpatient studies were assumed to best describe severe cases. Table 1 summarizes the algorithms used to determine the proportion of mild, moderate and severe cases occurring among children 0-59 mos of age; Table 1 also lists the source of duration and severity outcomes assumed to best describe each diarrhea category.

**Table 1:** Algorithms used to determine the total envelope of childhood and adult diarrhea

Envelope of Diarrhea	Severity	Algorithm (Data Source)^1^	Source of Duration & Severity Outcomes
Childhood Diarrhea	Mild	Proportion not seeking care (DHS)	Community-based studies
	
	Moderate	Proportion seeking care (DHS) Proportion outpatients without severe dehydration (MA)	Hospital outpatient studies
	
	Severe	Proportion seeking care (DHS) Proportion outpatients with severe dehydration (MA)	Hospital inpatient studies

Adult Diarrhea	Mild	Proportion community-based cases without dehydration (MA)	Community-based studies
	
	Moderate	Proportion community-based cases with any dehydration (MA)	Hospital outpatient studies
	
	Severe	Proportion community-based cases with any dehydration Proportion outpatients with severe dehydration (MA)	Hospital inpatient studies

^1^Data Sources: *DHS *Global average based on DHS report of diarrhea careseeking among children ≤ 35 mos; *MA *meta-analyses reported here

We also designed a model to describe the total envelope of diarrhea among individuals ≥ 16 years of age. We assumed that mild cases were comprised of cases occurring in the community without dehydration, and moderate cases were comprised of the proportion of community cases with any dehydration. In keeping with the assumptions made for the model describing the total envelope of diarrhea among children under-five, we used the proportion of outpatient cases presenting with severe dehydration to determine the percentage of moderate cases progressing to severe. We also assumed that community, hospital outpatient, and hospital inpatient studies best described the duration and severity profiles of mild, moderate, and severe cases, respectively (See Table 1).

## Results

### Systematic literature review

**Figure 1:**
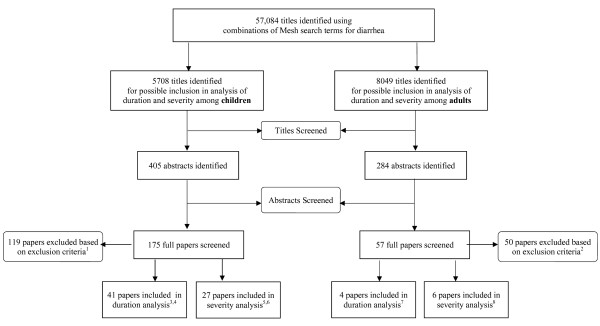
**Flow chart diagram of the systematic review process**. ^1^Main reasons for exclusion: report of one etiologic agent (n = 30); inadequate definition of diarrhea (n = 15); overlapping age categories (n = 17); no outcome of interest (n = 12); diarrhea recall beyond 2 weeks (n = 11); unclear/problematic methodology (n = 11); data in unusable form (n = 6); full episodes not followed (n = 6); not representative of general population (n = 4); combined data on hospital inpatients & outpatients (n = 7). ^2^Main reasons for exclusion: report of one etiologic agent (n = 20); overlapping age categories (n = 12); not representative of general population (n = 5); report of nosocomial infection (n = 3); unclear/problematic methodology (n = 3); diarrhea recall beyond 2 weeks (n = 4); inadequate definition of diarrhea (n = 1); no outcome of interest (n = 1); combined data on hospital inpatients & outpatients (n = 1). ^3^All studies reported data on children 0-59 mos of age; one study reported data on children 5-15 yrs of age. ^4^Study design: cohort (n = 27); RCT (n = 10); case-control (n = 4). Study location: community-based (n = 34); hospital-based (n = 7). ^5^All studies reported data on children 0-59 mos of age, only. ^6^Study design: cohort (n = 15); RCT (n = 5); case-control (n = 7). Study location: community-based (n = 10); hospital-based (n = 17). ^7^Study design: cohort (n = 2); RCT (n = 2). Study location: community-based (n = 2); hospital-based (n = 2). ^8^Study design: cohort (n = 4); RCT (n = 2). Study location: community-based (n = 2); hospital-based (n = 4).

**Figure 2:**
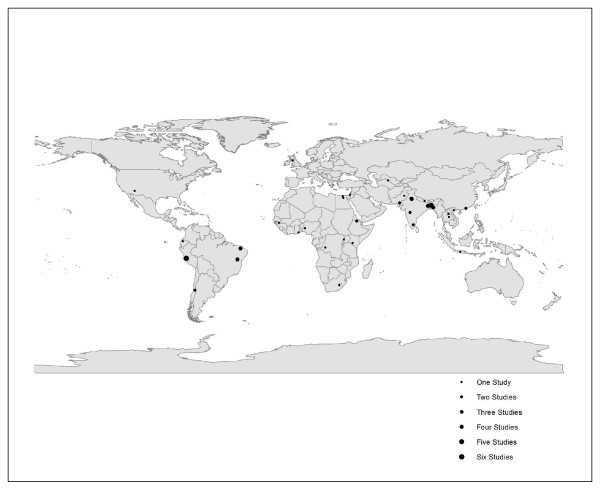
**Location of studies reporting diarrhea duration and severity outcomes for children and adults**. Locations of 61 unique studies; one study represented at 3 different locations.

The systematic literature search yielded 5708 titles for children (Figure 1), of which we identified 41 and 27 studies for inclusion in the analysis of duration [8-48] and severity [8,9,16,19,21,22,25,26,36,39,43,45,49-63], respectively. All included studies reported data on children 0-59 months of age. One duration study also reported outcomes on children 5-15 years of age [24]; no included studies reported data on severity outcomes among older children. Only one study was conducted in a high income country [48]. Of the 8049 titles identified for adults (Figure 1), we included four in the duration analysis [24,64-66], and six in the severity analysis [53,64-68]. All but two studies reported data from low- and middle-income countries (LMICs) [65,66]. Across analyses, we included 61 unique studies with study locations spanning across six WHO geographic regions (Figure 2).

### Duration of diarrheal episodes

Among children 0-59 months of age, the weighted mean duration was 4.3 days among cases assessed in the community and 8.4 days among hospital inpatients in LMICs (Table 2). The mean and median duration reported by the included study conducted in a high-income country were 2.2 days and 1 day, respectively.

**Table 2:** Estimates of diarrhea duration by age and country income-status

Duration Outcome	Study Design	Study Sites^1^ N	Combined Estimate % (95% CI)
**Low- and middle-income countries**			

**0-59 mos**			

**Weighted Mean^2^**	Community	29	4.3 (4.3-4.4)
	
	Hospital - Inpatient	2	8.4 (8.1-8.8)

**Weighted Median^2^**	Community	8	3.1 (3.0-3.2)
	
	Hospital - Inpatient	1	2.0 (-)

**Weighted Proportion Persistent^3^**	Community	12	4.9 (4.6-5.2)
	
	Hospital - Inpatient	4	11.1 (9.3-12.9)
	
	Hospital - Outpatient	1	15.4 (-)

**5-15 yrs**			

**Mean**	Community	1	2.7 (-)

**≥ 16 yrs**			

**Mean**	Community	1	3.0 (-)
	
	Hospital - Inpatient	1	2.6 (-)

**High-income countries**			

**0-59 mos**			

**Mean**	Community	1	2.2 (-)

**Median**	Community	1	1.0 (-)

**≥ 16 yrs**			

**Mean**	Community	1	2.6 (-)
	
	Hospital - Outpatient	1	4.6 (3.6-5.2)

^1^Individual studies may contribute more than one study site (N) to each estimate^2^Estimates for ≥ 2 study sites generated by Poisson regression model weighted by sample size^3^Estimates for ≥ 2 study sites generated by logistic regression model weighted by sample size

The sole study conducted among children 5-15 years of age reported a mean duration of 2.7 days for episodes assessed in the community (Table 2). The reported mean duration of episodes among adults ≥ 16 years of age was 3 days in the community and 2.6 days among hospital outpatients in LMICs (Table 2); in high-income countries, the mean duration of diarrhea was 2.6 days in the community and 4.6 days among outpatients.

### Severity of diarrheal illness

Any dehydration was the outcome most frequently cited as a measure of diarrhea severity among children under five. The weighted mean proportion of episodes categorized with any dehydration was 7.3% for episodes assessed in the community, 84.6% among hospital inpatients and 51.4% among hospital outpatients (Table 3). Any dehydration was also the most frequently reported measure of diarrhea severity among individuals ≥ 16 years of age (Table 4). The weighted mean proportion of episodes presenting with any dehydration was 92.8% for hospital inpatient studies, 30.1% for hospital outpatient studies, and 5.0% for community studies.

**Table 3:** Measures of severity of diarrhea among children 0-59 mos of age by study type

		Study Sites^1^ N	Weighted Mean^2^ % (95% CI)
**Community**			

**Dehydration**	Any	6	7.3 (6.6-8.0)

	Severe	1	10.9 (-)

**Vomiting**		10	18.1 (17.4-18.7)

**Bloody Stool**		9	8.7 (8.2-9.2)

**Stool Frequency^3^**	Frequency > 5 stools/day	3	13.4 (12.5-14.2)

	Frequency > 3 stools/day	2	57.5 (54.4-60.5)

	Mean Stool Frequency (stools/day)	3	4.99 (4.80-5.18)

**Hospital Outpatient**			

**Dehydration**	Any	3	51.4 (49.1-53.8)

	Mild	1	32.8 (-)

	Moderate	4	45.1 (43.5-46.7)

	Severe	2	1.4 (0.7-2.1)

**Vomiting**		2	55.7 (52.8-58.5)

**Stool Frequency^3^**	Low	1	63.4 (-)

	Medium	1	22.7 (-)

	High	1	14.0 (-)

	Frequency > 5 stools/day	1	55.1 (-)

	Frequency > 3 stools/day	3	84.4 (83.0-85.7)

**Hospital Inpatient**			

**Dehydration**	Any	7	84.6 (83.1-86.1)

	Mild	5	66.7 (63.4-70.0)

	Moderate	6	18.6 (16.2-20.9)

	Severe	10	8.1 (7.6-8.6)

**Vomiting**		2	58.3 (54.3-62.2)

**Bloody Stool**		1	16.2 (-)

**Stool Frequency^3^**	High	1	53.6 (-)

	Frequency > 5 stools/day	1	90.0 (-)

**Stool Volume**	Mean Stool Volume (kg/g/day)	1	52.6 (-)

^1^Individual studies (*n *= 27) may contribute more than one study site (N) to each estimate^2^Estimates for ≥ 2 study sites generated by logistic regression model weighted by sample size; mean stool frequency generated by Poisson regression model weighted by sample size^3^Low defined as 1-5 stools/day; medium as 6-9 stools/day; and high as ≥ 10 stools/day

**Table 4:** Measures of severity of diarrhea among individuals ≥ 16 yrs of age by country income-status

		Study Sites^1^ N	Weighted Mean^2^ % (95% CI)
**Low- and middle-income countries**			

**Community**			

**Dehydration**	Any	1	5.0 (-)

**Vomiting**		1	24.9 (-)

**Hospital Outpatient**			

**Dehydration**	Any	1	30.1 (-)

**Vomiting**		1	43.3 (-)

**Bloody Stool**		1	10.0 (-)

**Stool Frequency**	Mean Stool Frequency (stools/day)	1	8.1 (-)

**Hospital Inpatient**			

**Dehydration**	Any	2	92.8 (90.1-95.5)

	Mild	1	0 (-)

	Moderate	1	72.7 (-)

	Severe	2	40.3 (35.2-45.5)

**Vomiting**		1	83.0 (-)

**High-income countries**			

**Community**			

**Vomiting**		1	75.0 (-)

**Hospital Outpatient**			

**Vomiting**		1	25.9 (-)

^1^Individual studies (*n *= 4) may contribute more than one study site (N) to each estimate^2^Estimates for ≥ 2 study sites generated by logistic regression model weighted by sample size

### Diarrhea care-seeking practices

The DHS-based regional average and global median proportions of care-seeking for diarrhea among children under three years are presented in Table 5. Care-seeking for diarrhea was highest in North Africa/West Asia/Europe (41.0%) and lowest in South/Southeast Asia (31.0%) and West/Middle Africa (26.9%). The median global estimate of care-seeking for diarrhea among children under-five was 35.2%

**Table 5:** Global care-seeking practices for diarrhea episodes among children ≤ 36 mos of age in low- and middle-income countries

Region	Diarrhea Care-seeking (%)
West/Middle Africa*	26.9

South/East Africa**	39.1

North Africa/West Asia/Europe^^^	41.0

Central Asia^^^^	35.6

South/Southeast Asia^†^	31.0

Latin America/Caribbean^††^	34.8

**Global Median**	**35.2**

DHS[6] data sources: * Benin 2001, Burkina Faso 1998-99, Cameroon 1998, Chad 1996-97, Cote d'Ivoire 1998-99, Gabon 2000, Ghana 1998, Guinea 1999, Mali 2001, Mauritania 2000-01, Niger 1998, Nigeria 1999, Senegal 1997, Togo 1998. ** Comoros 1996, Eritrea 2002, Ethiopia 2000, Kenya 1998, Madagascar 1997, Malawi 2000, Mozambique 1997, Namibia 2000, Rwanda 2000, South Africa 19998, Tanzania 1999, Uganda 2000-01, Zambia 2001-02, Zimbabwe 1999. ^^ ^Armenia 2000, Egypt 2000, Jordan 2002, Turkey 1998, Yemen 1997. ^^^ ^Kazakhstan 1999, Kyrgz Rep. 1997, Turkmenistan 2000, Uzbekistan 1996. ^† ^Bangladesh 1999-00, Cambodia 2000, India 1998-99, Indonesia 1997, Nepal 2000, Philippines 1998, Vietnam 1997. ^†† ^Bolivia 1998, Brazil 1996, Colombia 2000, Dominican Rep. 2002, Guatemala 1998-99, Haiti 2000, Nicaragua 2001, Peru 2000.

### Estimating the total envelope of diarrhea

**Figure 3:**
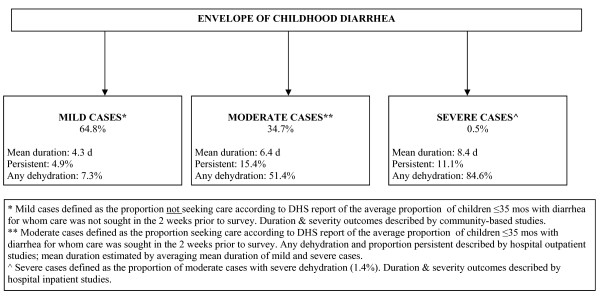
**Model of the duration and severity of diarrhea among children 0-59 mos of age**.

Figure 3 describes the total envelope of diarrhea morbidity among children 0-59 mos of age. Using care-seeking as a proxy for quantifying the proportion of diarrhea episodes which might be considered "mild", we estimate that 64.8% of episodes are mild and the remaining 35.2% of cases are moderate. Of these moderate cases, 1.4% will become severe (i.e. 0.5% of all cases). Using the data from community-based studies we estimate that mild cases last for 4.3 days. We estimate that 15.4% of moderate cases become persistent and 51.4% involve some dehydration. For severe cases, we applied the estimates derived from inpatient studies and thus assumed that severe cases endure 8.4 days with 84.6% resulting in dehydration. Since included outpatient studies did not report mean duration, we estimated the mean duration of moderate cases by averaging that of mild and severe cases; thus, moderate cases last approximately 6.4 days.

**Figure 4:**
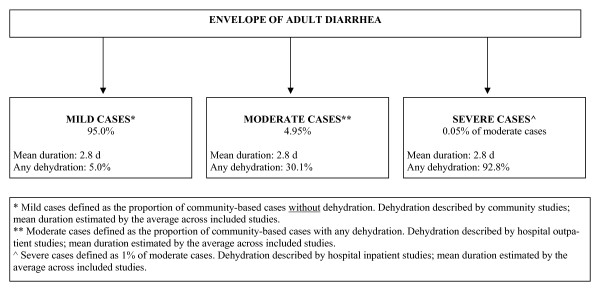
**Model of the duration and severity of diarrhea among individuals ≥ 16 yrs of age**.

Figure 4 describes the total envelope of diarrhea burden among individuals ≥ 16 years of age. Since severe dehydration was not reported by any of the outpatient studies included in this review, we determined the proportion of moderate cases progressing to severe among individuals ≥ 16 years of age by rounding down the proportion derived for children (1.4%); thus, of the 5% of cases characterized as moderate, 1% (i.e. 0.05% of all cases) evolve into severe episodes. Approximately 5% of mild, 30.1% of moderate and 92.8% of severe cases result in dehydration. Although we aimed to characterize the duration of mild, moderate, and severe cases using the averages reported by community, hospital inpatient, and hospital outpatient studies, respectively, mean duration was only reported by one included community study (3.0 days) and one included hospital inpatient study (2.6 days). We therefore averaged the duration reported by the two studies and applied this figure (2.8 days) to the duration of mild, moderate, and severe cases.

## Discussion

This systematic review is the first to describe the overall duration and severity of diarrhea among children and adults. To estimate the annual number of diarrhea episodes among children under-five by region, we applied the regional age-adjusted incidences of diarrhea among children 0-59 mos of age to the regional populations of children in that age group. The total number of diarrhea episodes, as well as the breakdown of mild, moderate, and severe episodes in each region, is presented in Table 6. By aggregating the regional numbers, we estimate that among the 1.67 billion diarrhea episodes occurring among children under-five globally per year 1.08 billion (64.8%) are mild; 580 million are moderate (34.7%); and 8 million (0.5%) are severe. By region, the burden of diarrhea is greatest in Africa and Asia; still, Table 7 illustrates that episodes occurring in Africa tend to be more serious in duration and severity outcomes.

**Table 6:** Regional breakdown of diarrhea episodes

GBD Region	Total # Diarrhea Episodes **0-59 mos**[71]*	Total # Mild Episodes	Total # Moderate Episodes	Total # Severe Episodes
African	448,755,780	290,793,745	155,718,256	2,243,779

Americas	220,278,200	142,740,274	76,436,535	1,101,391

Eastern Mediterranean	199,376,910	129,196,238	69,183,788	996,885

Europe	111,344,240	72,151,068	38,636,451	556,721

South East Asian	436,836,240	283,069,884	151,582,175	2,184,181

Western Pacific	254,965,120	165,217,398	88,472,897	1,274,826

**Total**	**1,671,556,490**	**1,083,168,606**	**580,030,102**	**8,357,782**

* Fischer Walker CL, Perin J, Aryee M, C. B-P, Black RE. Diarrhea incidence in low- and middle-income countries in 1990 and 2010. Submitted: BMC Public Health. 2011.

**Table 7:** Comparison of outcomes across Africa and Asia

Study Period	Outcome	Africa	Asia
1987-1991	Mean Duration^1^	4.4 (4.3-4.5)*	2.9 (2.8-3.0)*

1990-1994	Mild Dehydration^2^	69.8 (-)	65.6 (61.7-69.5)**

	Severe Dehydration^2^	30.2 (-)	7.2 (5.4-9.0)**

1992-1998	Any Dehydration^1^	77.4 (-)	1.9 (1.4-2.4)**

	Vomiting^1^	20.3 (19.0-21.6)**	19.4 (18.0-20.9)**

2000-2005	Mean Duration^1^	4.8 (4.6-5.1)*	3.4 (3.2-3.5)*

	Median Duration^1^	5.0 (-)	2.2 (2.1-2.3)*

*Weighted Mean/Median (95% CI); community-based studies, only**Weighted% (95% CI);

^1^Community-based studies, only

^2^Hospital-inpatient studies, only

Data were generated combining estimates for like study designs and outcomes in overlapping study years

The total envelope of diarrhea cases among individuals ≥ 16 years of age is comprised of approximately 430 million annual episodes of diarrhea [2]. We estimate that approximately 408.5 million episodes are mild (95%); 21.3 million are moderate (4.95%); and 0.2 million are severe (0.05%).

The dearth of studies reporting outcomes for children 5-15 years of age inhibits our ability to directly estimate the burden of diarrhea on this age group. However, a comparison of the mean duration of community-based cases seems to suggest that diarrhea outcomes among children aged 5-15 yrs are more similar to individuals ≥ 16 than to children under-five (Table 2). Accordingly, we applied the breakdown of mild, moderate, and severe cases estimated for individuals ≥ 16 to the total number of incident cases among children 5-15 years of age. We estimate that of the 200 million diarrheal episodes that occur per year among children 5-15 years of age [2], 190 million are mild (95%); 9.9 million are moderate (4.95%); and 0.1 million are severe (0.05%).

Our review is limited in that we cannot extrapolate our findings onto the duration and severity of diarrhea episodes occurring among HIV-infected individuals. Research comparing diarrhea morbidity and mortality between individuals of discrepant HIV status has shown that HIV increases susceptibility to infectious diseases, including diarrhea. Of the studies we identified, one excluded study observed that in addition to increased diarrhea incidence, HIV-infected children suffered higher duration and a larger proportion of persistent episodes as compared to uninfected children [69]. Moreover, the same study reported increased diarrhea-related mortality among HIV-infected children.

The proportion of moderate/severe episodes among children under-five, which was estimated using the proportion of episodes for which care was sought, is likely underestimated since appropriate care is sought in less than 30-50% of cases in resource poor settings [70]. Given that facility care is under-utilized, moderate/severe episodes among children under-five are also assessed in the community; thus, the duration and severity outcomes derived from community-based studies may not accurately describe mild episodes that do not require facility level care. However, the increasing gravity of duration and severity outcomes across community-based, hospital-inpatient, and hospital-outpatient studies, respectively, suggests that these estimates are plausible and although biased, they represent the best summary of available data.

## Conclusions

In this review we classified the total envelope of diarrheal episodes among individuals 0-59 mos, 5-15 years, and ≥ 16 years of age according to diarrhea duration and severity. This information is essential to accurately calculate the age-specific burden of disease and the appropriate allocation of DALYs. Given that the global burden of diarrhea consists of large numbers of episodes beyond those progressing to death, the estimated breakdown of mild, moderate, and severe episodes is increasingly important for informing policy decisions on global health. As programs increasingly scale-up and improve diarrhea treatment, especially among children under-five, estimates of diarrhea morbidity should be used to determine the most effective distribution of funds for interventions focused on diarrhea.

## Competing interests

The authors declare that they have no competing interests.

## Authors' contributions

LML conducted the systematic review, analysis and led the initial manuscript preparation. CLFW assisted with the analysis and the manuscript preparation. REB provided technical leadership and assisted with the interpretation of the analysis and the final manuscript preparation. All authors read and approved the final manuscript.

## Pre-publication history

The pre-publication history for this paper can be accessed here:

http://www.biomedcentral.com/1471-2458/12/276/prepub
