# Correction: Systematic review of diarrhea duration and severity in children and adults in low- and middle-income countries

**DOI:** 10.1186/1471-2458-12-832

**Published:** 2012-09-28

**Authors:** Laura M Lamberti, Christa L Fischer Walker, Robert E Black

**Affiliations:** 1 Department of International Health, Johns Hopkins Bloomberg School of Public Health, 615 N. Wolfe St, Baltimore, MD 21205, USA

## 

After the publication of this work [1], we became aware that we had incorrectly cited the number of diarrhea cases for 2005 among individuals 5–15 years and ≥ 16 years reported by Fischer Walker et al. [2]. We estimate that of the 2.115 billion episodes of diarrhea among individuals ≥ 16 years in 2005 [2], 2.009 billion episodes were mild (95%); 104.7 million were moderate (4.95%); and 1.1 million were severe (0.05%). Of the estimated 699.2 million diarrhea episodes among children 5–15 years of age in 2005 [2], an estimated 664.2 million were mild (95%); 34.6 million were moderate (4.95%); and 350,000 were severe (0.05%).

## Pre-publication history

The pre-publication history for this paper can be accessed here:

http://www.biomedcentral.com/1471-2458/12/832/prepub
